# Reduction of depressive symptoms among patients with inflammatory bowel disease treated with biological therapy: A cross sectional study

**DOI:** 10.1192/j.eurpsy.2021.901

**Published:** 2021-08-13

**Authors:** J. Zinkeviciute, R. Strumila, E. Dlugauskas, S. Brasiskiene, A. Kiziela, S. Ambrasas

**Affiliations:** 1 Medicine Faculty, Vilnius University, Vilnius, Lithuania; 2 Institute Of Clinical Medicine, Clinic Of Psychiatry, Vilnius University, Vilnius, Lithuania

**Keywords:** tumor necrosis factor alfa inhibitors, autoimmune depression, inflammatory bowel disease.

## Abstract

**Introduction:**

Previous studies suggest that one of the possible depression pathophysiological pathways is autoimmune inflammation increasing inflammatory mediators’ levels and thus affecting mood.

**Objectives:**

To compare depression and anxiety symptoms among inflammatory bowel disease patients receiving TNF-α inhibitors and those receiving treatment as usual (TAU).

**Methods:**

Instruments: Ulcerative colitis activity index, Crohn’s disease activity index, the subscale of neurovegetative symptoms of the Beck depression inventory, Hospital anxiety and depression scale. Active ulcerative colitis or Chron‘s disease patients not using antidepressants were included in the study and divided into an experimental group (receiving TNF-α inhibitors) and control group (receiving TAU).

**Results:**

46 patients’ data were analyzed. Between the experimental group and the control group, the disease activity index was not significantly different (Chron’s disease 3.54 ±4.20; ulcerative colitis 5.70 ±5.00; p > 0.05) as well as the mean scores of the neurovegetative depression symptoms subscale of the Beck depression inventory (2.52 experimental ±3.91 control; p > 0.05). The mean score of the hospital anxiety and depression scale were significantly different between both groups (5.22 ±8.13; p < 0.05). The mean anxiety subscale scores’ p=0,06, which shows trend for significance. The mean depressive subscale score was significantly different in the control group (1.43 ±2.65; p < 0.05).

**Conclusions:**

Patients treated with biological therapy experienced fewer depression symptoms than patients showing similar disease activity, but receiving TAU.

